# Integrating DNA methylation and microRNA biomarkers in sputum for lung cancer detection

**DOI:** 10.1186/s13148-016-0275-5

**Published:** 2016-10-19

**Authors:** Yun Su, HongBin Fang, Feng Jiang

**Affiliations:** 1 Department of Surgery, Jiangsu Province Hospital of Nanjing University of Chinese Medicine, 138 Xianlin Road, Nanjing, 210023 China; 2 Department of Epidemiology, University of Maryland School of Medicine, Baltimore, MD USA; 3 Department of Pathology, University of Maryland School of Medicine, Baltimore, MD USA

**Keywords:** Lung cancer, Sputum, MicroRNA, DNA methylation, Diagnosis

## Abstract

**Background:**

Abnormal microRNA (miRNA) expressions and promoter methylation of genes detected in sputum may provide biomarkers for non-small lung cancer (NSCLC). Here, we evaluate the individual and combined analysis of the two classes of sputum molecular biomarkers for NSCLC detection.

**Results:**

We analyze expression of 3 miRNAs (miR-21, miR-31, and miR-210) and methylation of 3 genes (*RASSF1A*, *PRDM14*, and *3OST2*), which were previously identified as potential biomarkers for NSCLC, in sputum of a set of 117 stage I NSCLC patients and 174 cancer-free smokers. The results are validated in a different set of 144 stage I NSCLC patients and 171 controls. The panel of 3 miRNA biomarkers has 81.5 % sensitivity and 85.9 % specificity; the panel of 3 methylation biomarkers displays 82.9 % sensitivity and 76.4 % specificity for NSCLC detection. Integrated analysis of 2 miRNAs (miR-31 and miR-210) and 2 genes (*RASSF1A* and *3OST2*) yields higher sensitivity (87.3 %) and specificity (90.3 %) compared with the individual panels of the biomarkers (*P* < 0.05). Combined analysis of all the 3 miRNAs and 3 genes does not have performance superior to that of the panel of 2 miRNAs and 2 genes (*P* > 0.05). The performance of combined use of the two classes of biomarkers was confirmed in the validation set.

**Conclusions:**

The integration of two different classes of biomarkers synergistically improves both the sensitivity and the specificity for the early detection of NSCLC.

**Electronic supplementary material:**

The online version of this article (doi:10.1186/s13148-016-0275-5) contains supplementary material, which is available to authorized users.

## Background

Lung cancer is the number one cancer killer worldwide [1]. Non-small cell lung cancer (NSCLC) accounts for approximately 85 % of all lung cancer cases. Tobacco smoking is the major cause of the disease. The overall 5-year survival rate for stage I NSCLC patients who are typically treated with surgery remains up to 83 %. In contrast, only 5–15 % and less than 2 % of patients with stage III and IV NSCLC are alive after 5 years [1]. These statistics provide the primary rationale to improve the early detection of NSCLC. Recently, a NCI-National Lung Screening Trail (NLST) showed that the early detection of lung cancer by using low-dose computed tomography (LDCT) could significantly reduce the mortality [2]. However, 25 % of smokers screened by LDCT have indeterminate pulmonary nodules (PNs), of which 95 % are lastly determined to be false positives. Given the high-false positive rate of LDCT, there is large number of referrals for invasive biopsies and expensive 2-year multiple follow-up examinations that carry their own morbidity and mortality. Therefore, it is clinically imperative to develop a noninvasive and cost-effective means that might be used alone or serve to supplement LDCT findings for precisely identifying early stage NSCLC.

Sputum is a noninvasively and easily accessible body fluid that contains respiratory epithelial cells exfoliated from the bronchial airways. Cytological study of sputum can identify morphological abnormalities of bronchial epitheliums and thus provides a noninvasive approach for lung cancer detection. However, sputum cytology has a poor sensitivity for detection of lung cancer at the early stage. It has been well accepted that NSCLC develops from a field defect characterized by an accumulation of molecular abnormalities resulted from repeated exposure of the airway of the smokers to the tobacco-related carcinogens [3]. Kadara et al. showed that the molecular alterations observed in the large bronchial airway might reflect the altered changes existed in lung tumors in the distal lung, regardless of the anatomic location relative to the tumors [4]. Furthermore, Spira et al. [5] demonstrated that analysis of the bronchial epitheliums of the airway of NSCLC patients could detect the lung tumor-related molecular changes and thus help diagnose lung cancer. Since sputum contains exfoliated bronchial epithelial cells from the lungs, examination of sputum might identify the molecular abnormalities in the large bronchial airways that reflected those existing in primary lung tumors [6]. Therefore, the analysis of sputum for the molecular changes may provide a noninvasive and cost-effective approach for lung cancer diagnosis.

CpG dinucleotides are in the promoter region of many genes, particularly tumor suppressor genes (TSGs). DNA methylation in the promoter region is frequently associated with “gene silencing” [7]. Aberrant promoter methylation can affect genes involved in cell-cycle control, DNA repair, cell adhesion, signal transduction, apoptosis, and cell differentiation [7]. These epigenetic changes are early events in carcinogenesis of NSCLC and thus show great promise as biomarkers for lung cancer early detection [8]. Various genes have been identified to display hypermethylation in lung tumor tissues as opposed to noncancerous tissues [6, 9]. Importantly, Belinsky et al. detected methylation of some TSGs in sputum up to 3 years prior to the clinical diagnosis of lung cancer [6, 9]. Hubers et al. recently demonstrated that DNA methylation analysis of a panel of 3 genes (*RASSF1A*, *PRDM14*, and *3OST2*) in sputum produced a sensitivity of 82 % and a specificity of 66 % for lung cancer detection [10]. Although the previous studies showed that the 3 genes used together provided the most promising sputum methylation biomarkers for early stage NSCLC, the sensitivity and specificity of the DNA methylation biomarkers are not sufficient to be used in the clinical settings for the early detection of NSCLC.

MicroRNAs (miRNAs) have important function in the regulation of gene expression in various biological processes [11]. Dysregulation of miRNAs plays crucial roles in tumorigenesis [11]. Specific over- or under-expressions of some miRNAs have been found to associate with lung tumor and thus open up a new field for molecular diagnosis of NSCLC. Furthermore, endogenous miRNAs are resistant to freeze-thaw action and stably exist in clinical samples, due to the small size and relative resistance to nucleases [12]. We have for the first time demonstrated that the miRNAs are reproducibly and specifically measurable in sputum by using quantitative reverse transcription-PCR (qRT-PCR) [13], thus providing a rationale for developing miRNAs as sputum biomarkers for NSCLC. In addition, using a microarray-based platform to profile expression of 818 mature miRNAs on NSCLC tissues and the paired normal lung tissues, we identified a set of 13 miRNAs (miRs-21, 31, 126, 139, 182, 200b, 205, 210, 375, 429, 486, and 708) that displayed dysregulation in NSCLC [14–16]. We further showed 10 of the 13 miRNAs (miRs-21, 31, 126, 182, 200b, 205, 210, 375, 486, and 708) whose abnormal expressions in sputum were related to lung cancer [14, 15]. Moreover, from the miRNAs, we identified a panel of 3 sputum miRNA biomarkers (miR-21, miR-31, and miR-210) with 82 % sensitivity and 86 % specificity for NSCLC detection [17]. Although showing promising, the panel of 3 sputum miRNA biomarkers also suffers from moderate sensitivity and specificity for the early detection of lung cancer.

Since NSCLC is a heterogeneous disease and develops from multifactorial molecular aberrations [4], the analysis of a single type of molecular changes (for example, either dysregulation of miRNAs or gene promoter hypermethylation) may not achieve the performance required to move forward for clinical application. Furthermore, because dysregulation of the miRNAs and promoter hypermethylation of TSGs have different and crucial roles in lung tumorigenesis via numerous cellular pathways, we hypothesize that integrating the miRNA and methylation biomarkers would have a synergistic effect for NSCLC detection. Here, we evaluate the individual and combined applications of the two classes of sputum molecular biomarkers for the early detection of lung cancer.

## Methods

### Patient cohorts

The study protocol was approved by the Institutional Review Board of Jiangsu Province Hospital of Nanjing University of Chinese Medicine. Written informed consent forms were obtained from all participants. Final diagnosis for NSCLC was made by using histopathologic examinations of biopsy and surgical tissue specimens. CT imaging was done by using a standard clinical protocol and read by radiologists. A positive result of CT was determined according to “the Fleischner Society-guidelines for management of small pulmonary nodules detected on CT scans.” The surgical pathologic staging was determined according to the TNM classification of the International Union Against Cancer with the American Joint Committee on Cancer and the International Staging System for Lung Cancer. Histopathologic classification was made according to the World Health Organization classification. Control individuals were smokers with CT-discovered PNs and 55–74 years old who had no prior history of any cancer. All control subjects remained cancer free for a minimum 2-year follow-up. The demographic and clinical characteristics of the recruited subjects, such as stage and histological diagnosis, smoking history, size of PN, and pulmonary functions, represented by forced expiratory volume in 1 s (FEV1)/forced vital capacity (FVC) were also collected.

### Sample collection, preparation, and sputum cytology

Sputum samples were collected from the participants before they revived any treatment as previously described [13–16, 18–27]. To reduce the percentage of oral epithelial cells in the sputum, subjects were asked to blow their nose, rinse their mouth, and swallow water to minimize contamination of squamous cells from postnasal drip and saliva. Sputum samples were then coughed in a sterile container and processed within 2 h. To further minimize oral squamous cell contamination, opaque or dense portions that looked different from saliva under the inverted microscope were selected using blunt forceps from expectorate. The samples were processed on ice in 4 volumes of 0.1 % dithiothreitol (Sigma-Aldrich, St. Louis, Mo) followed by 4 volumes of phosphate-buffered saline (PBS) (Sigma-Aldrich). The cell suspension was filtered through 45 μm nylon gauzes (BNSH Thompson, Scarborough, ON, Canada). Absolute cell numbers and cell viability were quantitated by using a hemacytometer with trypan blue. Two cytocentrifuge slides were prepared from aliquots of cell suspension by using a cytospin machine (Shandon, Pittsburgh, Pa) and were then stained with the Papanicolaou staining technique [28]. Furthermore, a 400 differential nonsquamous cell count was performed, and the differential cell count was expressed as the percentage of the total nonsquamous cells. A sputum sample was considered adequate if lung macrophages or Curschmann spirals were present on the slides [6, 28]. Positive sputum cytology for lung cancer comprised carcinoma in situ and invasive carcinoma.

### Analyzing DNA methylation in sputum by using qMSP

The isolation of DNA from sputum and the modification were performed as previously described [10]. Quantitative methylation specific PCR (qMSP) was done by using a Lightcycler system (Roche Applied Science, Mannheim, Germany) [10]. Hypermethylation markers for *RASSF1A*, *3OST2*, and *PRDM14* were selected, since the previous study [10] suggested that the 3 genes used together provided the most promising sputum methylation biomarkers for early stage NSCLC. Cycle threshold (Ct) values for each gene were determined. We normalized Ct values of the target genes in relation to that of *myogenic differentiation antigen 1* (*MYOD1*) [10, 29]. By using the formula: 2^∧^(Ct (*MYOD1*) − Ct (target gene)) × 100, we computed ratio value to decide the relative level of methylation of the genes in a given sample.

### Assessing expressions of the miRNAs in sputum by using qRT-PCR

We extracted RNA from sputum using a protocol established in our previous reports [13–16, 21, 22]. We evaluated the expressions of 3 miRNAs (miR-21, miR-31, and miR-210) by qRT-PCR with Taqman miRNA assays (Applied Biosystems, Foster City, CA) [13–16, 21, 22]. We calculated expression levels of the genes by using a comparative Ct method [13–17]. We normalized Ct values of the target miRNAs in relation to that of U6 and determined relative expression of a miRNA in a given sample using the equation 2 − ΔCt, where ΔCt = Ct (targeted ncRNA) − Ct (U6) [16, 17, 30]. Two interplate controls and one no-template control were carried along in each experiment. All experiments were performed for at least three times.

### Statistical analysis

Based on one sample with binomially distributed outcomes, we needed 45 NSCLC patients and 45 subjects with benign PNs in a training set at 5 % significant level with 80 % power to discover a panel of biomarkers. To estimate sample size of a testing set for the validation of the biomarkers, we used utilize area under the receiver-operator characteristic (ROC) curve (AUC) analysis. The AUC of H0 (the null hypothesis) was set at 0.5. H1 represented the alternative hypothesis. To have a high reproducibility with adequate precision, we required 60 subjects per group in the testing set. With this sample size, we would have 90 % power to detect an AUC of 0.75 at the 2 % significance level. Therefore, 117 stage I NSCLC patients and 174 cancer-free smokers in a training set and 144 stage I NSCLC patients and 171 controls in a testing set of this present study would provide enough statistical power to analyze and validate the biomarkers. We used a Wilcoxon rank-sum test to define the difference between case and control group and compute Spearman rank correlations among the molecular changes and with clinical-pathologic variables. We also used Pearson’s correlation analysis to assess the association between changes of the genes and demographic and clinical characteristics of the cancer cases or cancer-free controls. We applied AUC to evaluate sensitivity and specificity. We used the highest Youden’s J index (sum of sensitivity and specificity − 1) to set up corresponding cutoff value of each biomarker candidate [31]. We applied logistic regression [16] to identify composite panels of biomarkers that could distinguish NSCLC patients from control subjects and compare the diagnostic performances of the panels for lung cancer. The best panel of biomarkers was selected and subsequently tested in the validation set of the samples with the same thresholds. We used the McNemar to evaluate the complementary effect of the biomarkers to cytology for the detection of NSCLC.

## Results

### The characteristics of subjects and sputum samples

We enrolled 1707 smokers who could cough sputum. All of the sputum samples had deep lung macrophages or Curschmann’s spiral and thus were suitable for the cytological and molecular analysis [6, 32]. Among the participants, 828 were NSCLC patients and 879 were cancer-free smokers. Of the NSCLC cases, 261 had a diagnosis of stage I NSCLC, 246 had stage II, and 321 had stage III–IV NSCLC. Since we proposed to assess the individual and combined assessments of the two classes of molecule changes for lung cancer detection at the early stage, we only used sputum of the stage I NSCLC patients in this study. From the 879 cancer-free smokers, we selected 345 smokers with benign PNs as controls in this study. The 261 stage I lung cancer patients and 345 smokers with benign PNs were split into a training set and an internal testing set. The training set included 117 cancer cases and 174 smokers with benign PNs (Table 1). The 117 stage I lung cancer patients had a median age of 66.5 years. Ninety-three (79.5 %) were men. Sixty-three (53.8 %) NSCLC patients were diagnosed to have adenocarcinoma (AC), and 54 (46.2 %) have squamous cell carcinoma (SCC). All the lung cancer patients were smokers with a median of 45.8 pack-years of smoking. The 174 cancer-free controls were smokers with a median of 44.7 pack-years of smoking, of whom, 138 (79.3 %) were men. The cancer-free smokers had granulomatous inflammation (*n* = 84), nonspecific inflammatory changes (*n* = 51), or lung infections (*n* = 39). The testing (validation) set comprised 144 stage I NSCLC patients and 171 smokers with benign PNs (Table 2). The NSCLC patients had a median age of 66.3 years. One hundred fourteen (79.2 %) were men. Seventy-eight (54.2 %) NSCLC patients had AC, and 66 (45.8 %) had SCC. The NSCLC patients had a median of 44.4 pack-years of smoking. The 171 cancer-free subjects had a median age of 65.2 years and a median of 43.3 pack-years of smoking. One hundred thirty-five (78.9 %) were men. The cancer-free smokers had granulomatous inflammation (*n* = 99), nonspecific inflammatory changes (*n* = 51), or lung infections (*n* = 21). Because chronic obstructive pulmonary disease (COPD) could be present in about 60 % of lung cancer patients and impact molecular profiles in sputum, we used COPD as additional matching criteria for the smokers. COPD was classified using the Global Initiative for Chronic Obstructive Lung Disease (GOLD) criteria with GOLD 2 (50 % ≤ FEV1 < 80 % predicted) or greater indicative of airflow obstruction [33]. No significant difference of the age, FEV1/FVC or COPD, and smoking status was found between the NSCLC patients and the smokers with benign PNs (All *P* > 0.05), except size of PNs (Tables 1 and 2). Furthermore, there was no difference of the number of different cell types in sputum samples of the lung cancer patients versus the smokers with benign PNs (Additional file 1: Table S1).

**Table 1:** Characteristics of a training set of stage I NSCLC patients and smokers with benign pulmonary nodules

	NSCLC cases (*n* = 117)	Controls (*n* = 174)	*P* value
Age	66.53 (SD 10.98)	65.36 (SD 10.15)	0.34
Sex			0.36
Male	93	138	
Female	24	36	
Pack-years	45.83 (range, 6–148)	44.67 (range, 5–115)	0.34
FEV1/FVC	0.48–0.73	0.44–0.67	0.12
Nodule size (cm)	3.23 (SD, 1.62)	2.25 (SD, 1.04)	<0.01
Stage, all are stage I			
Histological type			
Adenocarcinoma (AC)	63		
Squamous cell carcinoma (SCC)	54		

*Abbreviations*: *NSCLC* non-small cell lung cancer, *SD* standard derivation, *FEV1/FVC* forced expiratory volume in 1 s/forced vital capacity

**Table 2:** Characteristics of a testing set of stage I NSCLC patients and smokers with benign pulmonary nodules

	NSCLC cases (*n* = 144)	Controls (*n* = 171)	*P* value
Age	66.32 (SD 12.68)	65.23 (SD 10.15)	0.33
Sex			0.39
Male	114	135	
Female	30	36	
Pack-years	44.38 (range, 6–169)	43.26 (range, 5–119)	0.35
FEV1/FVC	0.47–0.53	0.43–0.84	0.08
Nodule size (cm)	3.26 (SD, 1.38)	2.17 (SD, 0.98)	<0.01
Stage, all are stage I			
Histological type			
Adenocarcinoma (AC)	78		
Squamous cell carcinoma (SCC)	66		

*Abbreviations*: *NSCLC* non-small cell lung cancer, *SD* standard derivation, *FEV1/FVC* forced expiratory volume in 1 s/forced vital capacity

### Promoter methylation of the 3 genes in sputum had a significantly different level between NSCLC patients and smokers with benign PNs

The analysis of *RASSF1A*, *3OST2*, and *PRDM14* for the DNA hypermethylation was successfully performed in all the sputum samples. The methylation status of the 3 genes was significantly elevated in the NSCLC cases compared with the smokers with benign PNs in the training set (all *P* < 0.05). As shown in Table 3, the individual genes exhibited AUC values of 0.68–0.69, producing 45.3 to 59.3 % sensitivities and 77.3 to 86.2 % specificities for the early detection of NSCLC. The use of the 3 genes in combination generated a higher AUC (0.79) compared with each individual one (all *P* < 0.01) (Table 3). The DNA methylation of the 3 genes did not exhibit special association with a histological type of the NSCLC, size of PN, age, COPD status, and gender of the participants (all *P* > 0.05). Furthermore, the panel of 3 genes created 82.9 % sensitivity and 76.4 % specificity for the early detection of NSCLC. Our present study confirmed the previous finding [10] that the 3 genes could be potential methylation biomarkers for lung cancer detection.

**Table 3:** DNA hypermethylation of 3 genes in sputum of a training set of stage I NSCLC patients and smokers with benign pulmonary nodules

Genes	AUC (95 % CI)	Sensitivity (95 % CI)	Specificity (95 % CI)	*P* value
*RASSF1A*	0.68 (0.56 to 0.79)	45.3 (32.56 to 56.23)	86.2 (75.38 to 90.56)	<0.001
*3OST2*	0.69 (0.60 to 0.78)	49.3 (36.58 to 59.59)	84.5 (72.15 to 87.29)	<0.001
*PRDM14*	0.69 (0.58 to 0.79)	59.3 (47.40 to 68.73)	77.3 (65.5 to 85.16)	<0.001
Combined use of the 3 genes	0.79 (0.72 to 0.88)	82.9 (71.97 to 90.82)	76.4 (64.91 to 85.60)	<0.001

*Abbreviations*: *NSCLC* non-small cell lung cancer, *AUC* the area under receiver operating characteristic curve, *CI* confidence interval

### The 3 miRNAs displayed a considerably different level in sputum between NSCLC patients and smokers with benign PNs

The 3 miRNAs had a significantly higher expression level in sputum of the NSCLC patients compared with the control individuals (all *P* < 0.05). Furthermore, the individual miRNAs revealed AUC values of 0.76–0.84, resulting in 61.7 to 76.6 % sensitivities and 70.9 to 81.9 % specificities for the early detection of NSCLC (Table 4). Combined analysis of the 3 miRNAs produced 0.89 AUC (Table 4). Subsequently, the analysis of the 3 miRNAs together generated 81.5 % sensitivity and 85.9 % specificity for NSCLC detection. The expression of miR-21 in sputum was closely associated with AC (*P* < 0.05), whereas miR-210 was related to SCC (*P* < 0.05). The changes of the miRNAs were associated with size of PNs (*P* < 0.05). However, overall, the panel of 3 sputum miRNA biomarkers did not show special association with a histological type of the NSCLC, size of PN, age, COPD status, and gender of the participants (all *P* > 0.05). The results generated from this current study confirmed our previous discovery [17] that the 3 miRNAs would be potential sputum biomarkers for lung cancer.

**Table 4:** The expression of 3 miRNAs in sputum of a training set of stage I NSCLC patients and smokers with benign pulmonary nodules

miRNAs	AUC (95 % CI)	Sensitivity (95 % CI)	Specificity (95 % CI)	*P* value
miR-31	0.76 (0.70 to 0.83)	61.66 (55.35 to 67.68)	81.86 (76.23 to 86.83)	<0.001
miR-21	0.80 (0.72 to 0.86)	79.47 (72.38 to 82.94)	70.98 (65.48 to 77.46)	<0.001
miR-210	0.84 (0.78 to 0.90)	76.58 (68.35 to 80.13)	81.16 (75–23.37 to 86.18)	<0.001
Combined use of 3 miRNAs	0.89 (0.85 to 0.94)	81.48 (68.5 to 90.75)	85.91 (72.41 to 94.25)	<0.001

*Abbreviations*: *AUC* the area under receiver operating characteristic curve, *CI* confidence interval

### Integrated analysis of 2 miRNA and 2 DNA methylation biomarkers in sputum has a synergistic effect for lung cancer early detection

**Fig. 1:**
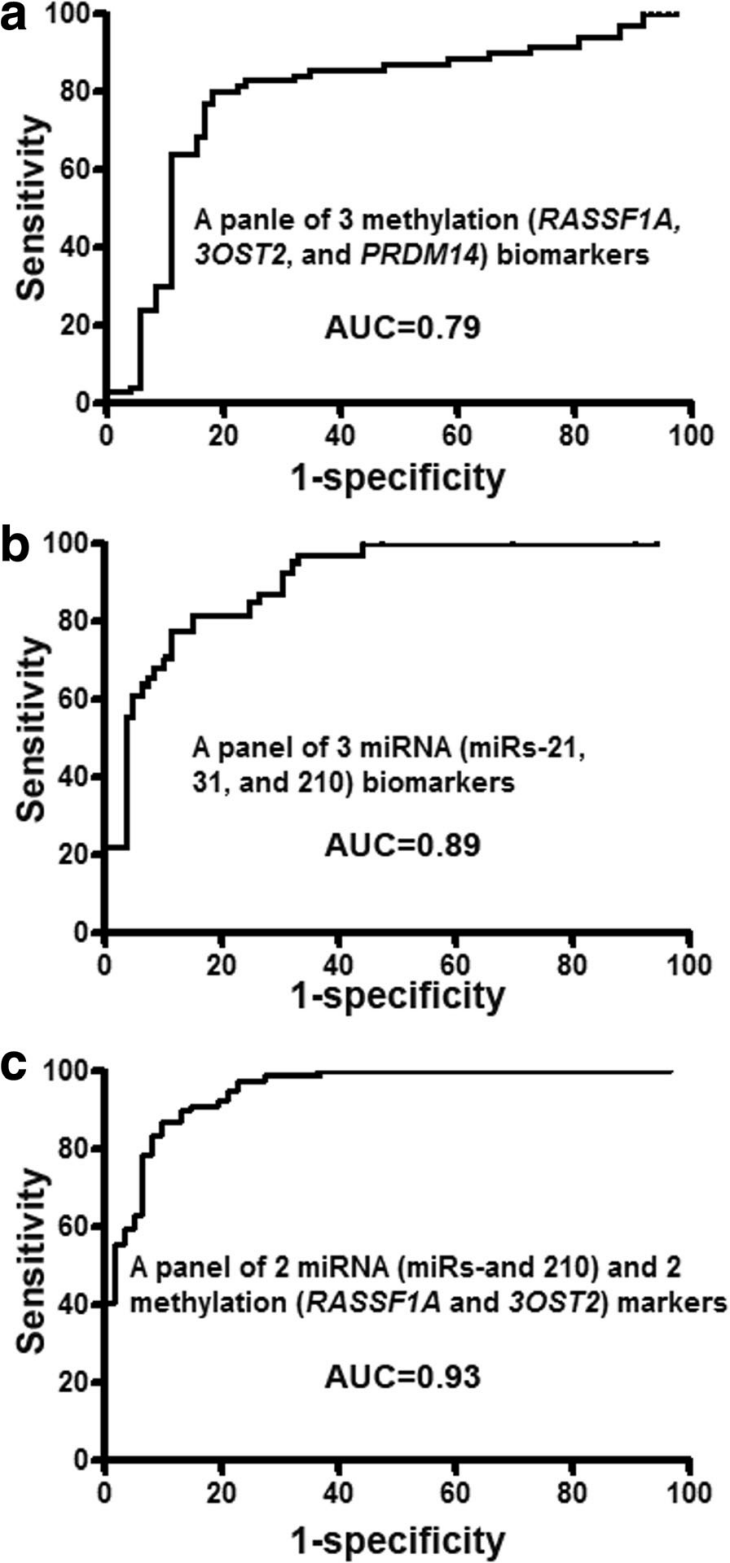
Combined analysis of miRNA and DNA methylation biomarkers in sputum has a synergistic effect for lung cancer detection. **a** ROC curve of a panel of 3 miRNA biomarkers (miR-21, miR-31, and miR-210) shows an AUC of 0.89 for differentiating NSCLC patients from smokers with benign pulmonary nodules in terms of sensitivity and specificity. **b** A panel of 3 DNA methylation biomarkers (*RASSF1A*, *3OST2*, and *PRDM14*) creates an AUC of 0.79 for distinguishing NSCLC patients from the cancer-free smokers. **c** The integration of 2 miRNAs (miR-31 and miR-210) and 2 TSGs (*RASSF1A* and *3OST2*) yields 0.93 AUC, which is statistically higher than that of the panel of 3 miRNA biomarkers and the panel of 3 methylation biomarkers (*P* < 0.05)

We used logistic regression models with constrained parameters as in least absolute shrinkage and selection operator (LASSO) and AUCs to determine performance of different patterns of combining the 3 miRNA and 3 DNA methylation biomarkers for lung cancer detection. From the 6 genes, 2 miRNAs (miR-31 and miR-210) and 2 genes (*RASSF1A* and *3OST2*) were selected as the best biomarkers (all *P* < 0.001). Incorporated use of the 4 biomarkers produced a higher AUC (0.93) (Fig. 1), as compared with the panel of 3 miRNA biomarkers (0.89) or the panel of methylation biomarkers (0.79) used alone (*P* < 0.05). Furthermore, the use of the 4 biomarkers together generated 87.3 % sensitivity and 90.4 % specificity (Table 5). As a result, the integration of 2 miRNAs (miR-31 and miR-210) and 2 TSGs (*RASSF1A* and *3OST2*) yielded higher sensitivity and specificity compared with the panel of 3 miRNA biomarkers and the panel of 3 methylation biomarkers used alone (all *P* < 0.05) (Table 5). Furthermore, the combined use of all the 6 biomarkers (3 miRNA and 3 methylation biomarkers) did not produce higher sensitivity and specificity compared with the panel of the 4 biomarkers (2 miRNAs and 2 genes) (*P* > 0.05). In addition, Pearson’s correlation analysis indicated that the estimated correlations among levels of the 4 molecular biomarkers were very low (all *P* > 0.05), implying that the integration of the different classes of biomarkers might have complementary classification. Moreover, sputum cytology had 46.2 % sensitivity and 89.7 % specificity. The combined use of the 4 sputum biomarkers had a higher sensitivity (*P* < 0.01) and a similar specificity compared with sputum cytology (*P* = 0.46). However, the addition of the cytology study in the biomarker panel did not improve the diagnostic efficiency for lung cancer detection. Overall, the panel of the 4 biomarkers had no special association with a histological type of the NSCLC, age, COPD status, and gender of the participants (all *P* > 0.05). In addition, the combined use of the 4 biomarkers showed a similar accuracy for the detection of NSCLC in PNs <10 mm versus PNs >10 mm.

**Table 5:** The performance of individual and combined applications of the two classes of sputum biomarkers for lung cancer detection

Biomarkers	AUC (95 % CI)	Sensitivity (95 % CI)	Specificity (95 % CI)	*P* value
The panel of the 3 miRNA biomarkers	0.89 (0.82 to 0.93)	81.50 (68.57 to 90.75)	85.91 (72.41 to 93.25)	<0.001
The panel of the 3 DNA methylation biomarkers	0.79 (0.72 to 0.87)	82.9 (71.97 to 90.82)	76.43 (64.91 to 85.60)	<0.001
Combined analysis of the two classes of sputum biomarkers	0.93 (0.89 to 0.96)	87.34 (77.95 to 93.76)	90.35 (80.12 to 96.37)	<0.001

*Abbreviations*: *NSCLC* non-small cell lung cancer, *AUC* the area under receiver operating characteristic curve, *CI* confidence interval

### Validating the synergistic effect of combined application of the miRNA and DNA methylation biomarkers for lung cancer detection

The optimized panel of the 4 sputum biomarkers consisting of 2 miRNAs with elevated expression and 2 genes with DNA methylation was validated in a testing cohort in a blinded fashion using the thresholds established in the above training set. The biomarker panel had 87.5 % sensitivity and 89.5 % specificity for lung cancer detection. Furthermore, sputum cytology showed 45.8 % sensitivity and 90.1 % specificity. The 4 biomarkers used in combination displayed a higher sensitivity (*P* < 0.01) and a similar specificity (*P* = 0.45) than did sputum cytology. Therefore, the results created from the validation set of samples confirmed the potential of integrated analysis of the two classes of molecular changes as a sputum assay for NSCLC detection.

## Discussion

Sputum is the most easily and noninvasively obtained clinical sample containing bronchial epithelial cells exfoliated from the lungs. Molecular analysis of sputum for lung cancer detection would be practically useful when diagnostic biomarkers are appropriately identified. The analysis of DNA methylation of a panel of 3 genes (*RASSF1A*, *3OST2*, and *PRDM14*) in sputum produced a sensitivity of 82 % and a specificity of 66 % for lung cancer detection [10]. The 3 genes have been shown as the most capable methylation biomarkers in sputum for the diagnosis of lung cancer at the early stage [10]. Furthermore, we have identified a panel of 3 sputum miRNA biomarkers (miR-21, miR-31, and miR-210) with 82 % sensitivity and 86 % specificity for NSCLC [17]. This present study demonstrates that the diagnostic performance of the sputum molecular biomarkers for lung cancer could be validated in a different set of sputum samples. Furthermore, the biomarkers developed in White Americans and African Americas are confirmed in a geographically independent cohort (Chinese population) and further imply the usefulness of the methylation and miRNA-based biomarkers for NSCLC detection.

Since lung cancer is a heterogeneous disease featuring field defects in the airway of smokers, a single class of biomarkers might not achieve the sensitivity and specificity required to move forward for clinically detecting NSCLC. Indeed, although exhibiting promising, the use of either the miRNA biomarker panel or the methylation biomarker panel has limited diagnostic value, due to the moderate sensitivity (81.5–82.9 %) and specificity (76.4–85.9 %). DNA promoter hypermethylation play crucial roles in the development and progression of cancer through transcriptionally silencing TSGs [3]. In contrast, miRNAs directly repress cancer-associated genes through binding to sites within coding and untranslated regions of mRNAs of the targets and hence contribute to tumorigenesis [11]. Therefore, the miRNAs and DNA methylations have highly and actively different functions in carcinogenesis. Given the heterogeneous nature of lung cancer and the numerous cellular pathways involved, we hypothesize that integrating the miRNA and DNA methylation biomarkers may improve the performance of the sputum assay for lung cancer detection. Our current data suggests that the combined analysis of the 2 miRNA and 2 methylation biomarkers yields a higher diagnostic performance compared with a single type of the molecular biomarkers used alone. Furthermore, the correlations among the changes of the miRNAs and promoter methylation of the TSGs are very low, supporting that the diagnostic vales of the two classes of molecular alterations could be complementary to each other. Therefore, the observation confirms our hypothesis. In addition, the finding that the panel of sputum biomarkers was not associated with a particular histologic type of NSCLC substantiates the utility for predicting lung cancer. Moreover, the combined use of the miRNA and DNA methylation biomarkers had a comparable diagnostic performance NSCLC in PNs <10 mm versus PNs >10 mm. The integrated biomarkers would be practical to use, since accurately identifying NSCLC among individuals with PNs <10 mm is one of the most clinically challenging [2].

Dysregulation of miR-31 was observed in various types of cancer disease, including colorectal, head-and-neck, and esophageal tumors [34]. The examination of serum miR-210 expressions could help identify diffuse large B cell lymphoma and pancreatic and lung cancers [35–37]. Epigenetic inactivation of the *RASSF1* promoter region is one of the earliest molecular events in lung tumorigenesis [38, 39]. Furthermore, methylation of *RASSF1A* is one of the major biomarker with increased risk of lung cancer [40]. *3OST2* undergoes frequent promoter methylation in various tumors [41–43], including lung cancer [10]. This study extends the previous findings by demonstrating that integrated analysis of the 4 molecular changes could be a potentially useful and efficient approach for lung cancer early detection.

The study does have some limitations. (i) Ideal biomarkers should be very highly sensitive and specific for NSCLC detection at the early stage. However, the combined use of the two types of molecular biomarkers, whereas promising, does not possess the required diagnostic discrimination for routine clinical application. In the future, we should identify additional miRNA or DNA methylation biomarkers that can be added to the current ones so that the diagnostic efficacy of the sputum assay could be improved. Furthermore, we have shown that the assessment of numerical DNA copy-number changes of genes or chromosomal aneusomy in sputum could help diagnose early stage lung cancer [24–27, 44–46]. Integrated assessment of the miRNAs and DNA methylation with the genomic and chromosomal changes would also improve the early detection of lung cancer. (ii) The objective of this project is to evaluate the individual and combined applications of the two classes of sputum biomarkers for the early detection of lung cancer. We do not test the biomarkers in the sputum samples of patients diagnosed with advanced stage of NSCLC. However, we will analyze the biomarkers in patients with different stages of NSCLC to determine if there is correlation of the biomarkers with stage of lung cancer and whether the biomarkers could be used to predict outcome of the disease. (iii) The early detection of NSCLC using LDCT followed by appropriate treatments can significantly reduce lung cancer mortality in smokers [2]. LDCT is now recommended for lung cancer screening in smokers. Yet LDCT has a low specificity for the early detection of lung cancer, presenting a major clinical challenge [2]. The development of the biomarkers for specifically identifying NSCLC in a LDCT screening positive setting will reduce lung cancer mortality by sparing smokers with benign PNs from invasive and expense multiple follow-up examinations and facilitating effective treatments to be instantly initiated for NSCLC [2]. However, cases and controls used in this study were recruited from the hospital-based patients with CT-discovered PNs. The participants might not well representative of the smokers in LDCT screening setting for lung cancer. We will perform a prospective trial to determine if the analysis of the sputum biomarkers could be used as an effective high-throughput screening for specifically identifying NSCLC in a LDCT screening positive setting among smokers. (iv) The number of females enrolled in the study is significantly lower than the number of males. In the future, we will perform a prospective study to recruit appropriate number of females and further evaluate if a gender effect can be observed using these biomarkers. Furthermore, the results were based on a Chinese population with PNs. We will investigate if there are ethnicity-specific associations of the panel of multifaceted biomarkers with other ethnic groups for the early detection of lung cancer.

## Conclusions

Given the heterogeneous nature of NSCLC that develops from multifactorial molecular aberrations, we have for the first time demonstrated that the integration of DNA methylation and miRNA biomarkers could provide a more efficient approach for the early detection of lung cancer. Nonetheless, a large multicenter clinical project to further validate the full utility of the combined approach is required before the biomarkers could be adopted in routine clinical setting.

## Additional file

Additional file 1: Table S1. Differential cell percentages of stage I non-small cell lung (NSCLC) patients and smokers with benign pulmonary nodules. (XLS 25 kb)

## References

[1] Cancer facts & figures 2012. American Cancer Society (ACS). Journal of Consumer Health on the Internet. 2012; 16:366–367.

[2] Aberle DR, Adams AM, Berg CD, Black WC, Clapp JD, Fagerstrom RM (2011). Reduced lung-cancer mortality with low-dose computed tomographic screening. N Engl J Med.

[3] Belinsky SA (2004). Gene-promoter hypermethylation as a biomarker in lung cancer. Nat Rev Cancer.

[4] Kadara H, Wistuba II (2012). Field cancerization in non-small cell lung cancer: implications in disease pathogenesis. Proc Am Thorac Soc.

[5] Brody JS, Spira A (2006). State of the art. Chronic obstructive pulmonary disease, inflammation, and lung cancer. Proc Am Thorac Soc.

[6] Belinsky SA, Liechty KC, Gentry FD, Wolf HJ, Rogers J, Vu K (2006). Promoter hypermethylation of multiple genes in sputum precedes lung cancer incidence in a high-risk cohort. Cancer Res.

[7] Leonhardt H, Cardoso MC (2000). DNA methylation, nuclear structure, gene expression and cancer. J Cell Biochem Suppl.

[8] Belinsky SA, Nikula KJ, Palmisano WA, Michels R, Saccomanno G, Gabrielson E (1998). Aberrant methylation of p16 (INK4a) is an early event in lung cancer and a potential biomarker for early diagnosis. Proc Natl Acad Sci U S A.

[9] Belinsky SA, Palmisano WA, Gilliland FD, Crooks LA, Divine KK, Winters SA (2002). Aberrant promoter methylation in bronchial epithelium and sputum from current and former smokers. Cancer Res.

[10] Hubers AJ, Heideman DA, Burgers SA, Herder GJ, Sterk PJ, Rhodius RJ (2015). DNA hypermethylation analysis in sputum for the diagnosis of lung cancer: training validation set approach. Br J Cancer.

[11] Croce CM, Calin GA (2005). miRNAs, cancer, and stem cell division. Cell.

[12] Mitchell PS, Parkin RK, Kroh EM, Fritz BR, Wyman SK, Pogosova-Agadjanyan EL (2008). Circulating microRNAs as stable blood-based markers for cancer detection. Proc Natl Acad Sci U S A.

[13] Xie Y, Todd NW, Liu Z, Zhan M, Fang H, Peng H (2010). Altered miRNA expression in sputum for diagnosis of non-small cell lung cancer. Lung Cancer.

[14] Xing L, Todd NW, Yu L, Fang H, Jiang F (2010). Early detection of squamous cell lung cancer in sputum by a panel of microRNA markers. Mod Pathol.

[15] Yu L, Todd NW, Xing L, Xie Y, Zhang H, Liu Z (2010). Early detection of lung adenocarcinoma in sputum by a panel of microRNA markers. Int J Cancer.

[16] Shen J, Liao J, Guarnera MA, Fang H, Cai L, Stass SA (2014). Analysis of MicroRNAs in sputum to improve computed tomography for lung cancer diagnosis. J Thorac Oncol.

[17] Xing L, Su J, Guarnera MA, Zhang H, Cai L, Zhou R (2015). Sputum microRNA biomarkers for identifying lung cancer in indeterminate solitary pulmonary nodules. Clin Cancer Res.

[18] Romeo MS, Sokolova IA, Morrison LE, Zeng C, Baron AE, Hirsch FR (2003). Chromosomal abnormalities in non-small cell lung carcinomas and in bronchial epithelia of high-risk smokers detected by multi-target interphase fluorescence in situ hybridization. J Mol Diagn.

[19] Varella-Garcia M, Kittelson J, Schulte AP, Vu KO, Wolf HJ, Zeng C (2004). Multi-target interphase fluorescence in situ hybridization assay increases sensitivity of sputum cytology as a predictor of lung cancer. Cancer Detect Prev.

[20] Yu L, Shen J, Mannoor K, Guarnera M, Jiang F (2014). Identification of ENO1 as a potential sputum biomarker for early-stage lung cancer by shotgun proteomics. Clin Lung Cancer.

[21] Li N, Ma J, Guarnera MA, Fang H, Cai L, Jiang F (2014). Digital PCR quantification of miRNAs in sputum for diagnosis of lung cancer. J Cancer Res Clin Oncol.

[22] Anjuman N, Li N, Guarnera M, Stass SA, Jiang F (2013). Evaluation of lung flute in sputum samples for molecular analysis of lung cancer. Clin Transl Med.

[23] Jiang F, Todd NW, Li R, Zhang H, Fang H, Stass SA (2010). A panel of sputum-based genomic marker for early detection of lung cancer. Cancer Prev Res (Phila).

[24] Jiang F, Todd NW, Qiu Q, Liu Z, Katz RL, Stass SA (2009). Combined genetic analysis of sputum and computed tomography for noninvasive diagnosis of non-small-cell lung cancer. Lung Cancer.

[25] Katz RL, Zaidi TM, Fernandez RL, Zhang J, He W, Acosta C (2008). Automated detection of genetic abnormalities combined with cytology in sputum is a sensitive predictor of lung cancer. Mod Pathol.

[26] Qiu Q, Todd NW, Li R, Peng H, Liu Z, Yfantis HG (2008). Magnetic enrichment of bronchial epithelial cells from sputum for lung cancer diagnosis. Cancer.

[27] Li R, Todd NW, Qiu Q, Fan T, Zhao RY, Rodgers WH (2007). Genetic deletions in sputum as diagnostic markers for early detection of stage I non-small cell lung cancer. Clin Cancer Res.

[28] Saccomanno G, Saunders RP, Archer VE, Auerbach O, Kuschner M, Beckler PA (1965). Cancer of the lung: the cytology of sputum prior to the development of carcinoma. Acta Cytol.

[29] Hubers AJ, van der Drift MA, Prinsen CF, Witte BI, Wang Y, Shivapurkar N (2014). Methylation analysis in spontaneous sputum for lung cancer diagnosis. Lung Cancer.

[30] Su J, Liao J, Gao L, Shen J, Guarnera MA, Zhan M (2015). Analysis of small nucleolar RNAs in sputum for lung cancer diagnosis. Oncotarget.

[31] Bohning D (2015). Youden’s index and the likelihood ratio positive in diagnostic testing. Methods Inf Med.

[32] Vestbo J, Hurd SS, Agusti AG, Jones PW, Vogelmeier C, Anzueto A (2013). Global strategy for the diagnosis, management, and prevention of chronic obstructive pulmonary disease: GOLD executive summary. Am J Respir Crit Care Med.

[33] Wang S, Hu J, Zhang D, Li J, Fei Q, Sun Y (2014). Prognostic role of microRNA-31 in various cancers: a meta-analysis. Tumour Biol.

[34] Chang W, Lee CY, Park JH, Park MS, Maeng LS, Yoon CS (2013). Survival of hypoxic human mesenchymal stem cells is enhanced by a positive feedback loop involving miR-210 and hypoxia-inducible factor 1. J Vet Sci.

[35] Lawrie CH, Gal S, Dunlop HM, Pushkaran B, Liggins AP, Pulford K (2008). Detection of elevated levels of tumour-associated microRNAs in serum of patients with diffuse large B-cell lymphoma. Br J Haematol.

[36] Papaconstantinou IG, Manta A, Gazouli M, Lyberopoulou A, Lykoudis PM, Polymeneas G (2013). Expression of microRNAs in patients with pancreatic cancer and its prognostic significance. Pancreas.

[37] Hesson LB, Cooper WN, Latif F (2007). Evaluation of the 3p21.3 tumour-suppressor gene cluster. Oncogene.

[38] Dammann R, Li C, Yoon JH, Chin PL, Bates S, Pfeifer GP (2000). Epigenetic inactivation of a RAS association domain family protein from the lung tumour suppressor locus 3p21.3. Nat Genet.

[39] Donninger H, Vos MD, Clark GJ (2007). The RASSF1A tumor suppressor. J Cell Sci.

[40] Liu J, Shworak NW, Sinay P, Schwartz JJ, Zhang L, Fritze LM (1999). Expression of heparan sulfate D-glucosaminyl 3-O-sulfotransferase isoforms reveals novel substrate specificities. J Biol Chem.

[41] Chen H, Zhang C, Sheng Y, Yao S, Liu Z, Zhang T (2015). Frequent SOCS3 and 3OST2 promoter methylation and their epigenetic regulation in endometrial carcinoma. Am J Cancer Res.

[42] Hubers AJ, Brinkman P, Boksem RJ, Rhodius RJ, Witte BI, Zwinderman AH (2014). Combined sputum hypermethylation and eNose analysis for lung cancer diagnosis. J Clin Pathol.

[43] Katz RL, He W, Khanna A, Fernandez RL, Zaidi TM, Krebs M (2010). Genetically abnormal circulating cells in lung cancer patients: an antigen-independent fluorescence in situ hybridization-based case-control study. Clin Cancer Res.

[44] Li R, Wang H, Bekele BN, Yin Z, Caraway NP, Katz RL (2006). Identification of putative oncogenes in lung adenocarcinoma by a comprehensive functional genomic approach. Oncogene.

[45] Jiang F, Caraway NP, Nebiyou Bekele B, Zhang HZ, Khanna A, Wang H (2005). Surfactant protein A gene deletion and prognostics for patients with stage I non-small cell lung cancer. Clin Cancer Res.

[46] Jiang F, Yin Z, Caraway NP, Li R, Katz RL (2004). Genomic profiles in stage I primary non-small cell lung cancer using comparative genomic hybridization analysis of cDNA microarrays. Neoplasia.

